# The Expression Regulation and Biological Function of Autotaxin

**DOI:** 10.3390/cells10040939

**Published:** 2021-04-19

**Authors:** Xiaotian Zhang, Mengmiao Li, Nan Yin, Junjie Zhang

**Affiliations:** The Key Laboratory of Cell Proliferation and Regulation Biology, Ministry of Education, Institute of Cell Biology, College of Life Sciences, Beijing Normal University, Beijing 100875, China; xiaotianzhang@bnu.edu.cn (X.Z.); 201831200013@mail.bnu.edu.cn (M.L.); Yinnan@mail.bnu.edu.cn (N.Y.)

**Keywords:** Autotaxin (ATX), lysophosphatidic acid (LPA), ATX-LPA axis, cancer

## Abstract

Autotaxin (ATX) is a secreted glycoprotein and functions as a key enzyme to produce extracellular lysophosphatidic acid (LPA). LPA interacts with at least six G protein-coupled receptors, LPAR1-6, on the cell membrane to activate various signal transduction pathways through distinct G proteins, such as Gi/0, G12/13, Gq/11, and Gs. The ATX-LPA axis plays an important role in physiological and pathological processes, including embryogenesis, obesity, and inflammation. ATX is one of the top 40 most unregulated genes in metastatic cancer, and the ATX-LPA axis is involved in the development of different types of cancers, such as colorectal cancer, ovarian cancer, breast cancer, and glioblastoma. ATX expression is under multifaceted controls at the transcription, post-transcription, and secretion levels. ATX and LPA in the tumor microenvironment not only promote cell proliferation, migration, and survival, but also increase the expression of inflammation-related circuits, which results in poor outcomes for patients with cancer. Currently, ATX is regarded as a potential cancer therapeutic target, and an increasing number of ATX inhibitors have been developed. In this review, we focus on the mechanism of ATX expression regulation and the functions of ATX in cancer development.

## 1. ATX, a Lyso-PLD that Catalyzes Extracellular LPA Production

**Figure 1:**
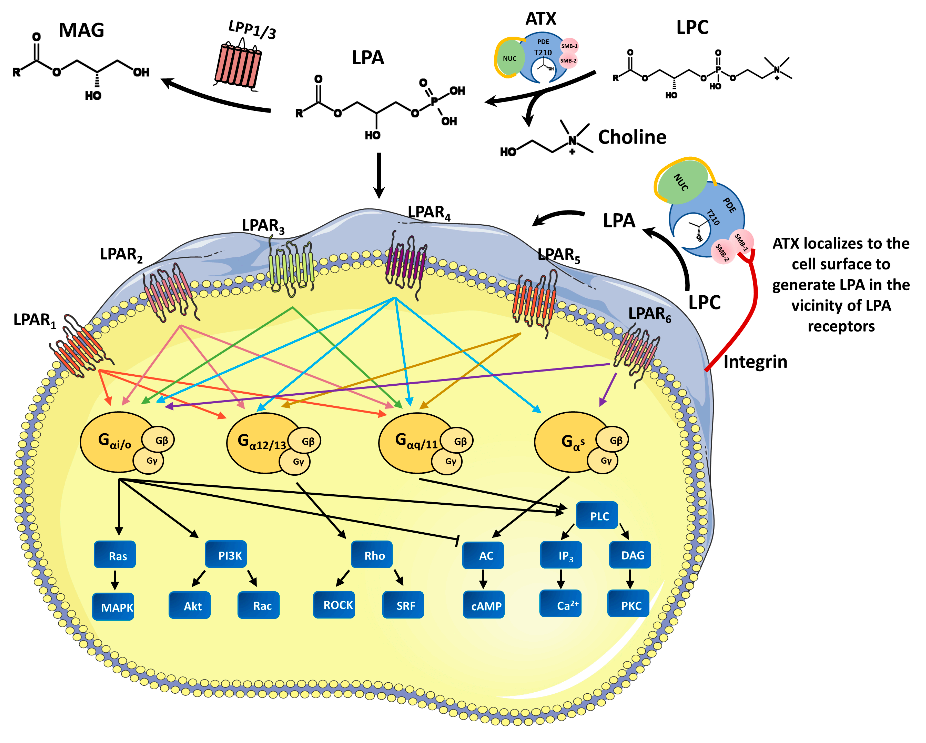
ATX-LPA-LPA receptor signaling axis and LPA delivery on the surface of target cell. LPA is mainly synthesized via the ATX-mediated hydrolysis of lysophosphatidylcholine (LPC) and degraded to monoacylglycerol (MAG) through LPP1/3. ATX protein is composed of two N-terminal somatomedin B (SMB)-like domains, a central phosphodiesterase (PDE) domain, and a C-terminal nuclease (NUC)-like domain. The SMB2 domain enables ATX to bind with cell surface integrin, which achieves a highly efficient delivery of LPA to its receptors. When it binds to LPAR_1-6_, LPA induces different downstream signal cascades, including Rho, phospholipase C (PLC), mitogen-activated protein kinase (MAPK), phosphatidylinositide 3-kinase (PI3K), and adenylate cyclase (AC), through different G proteins.

Autotaxin (ATX), a secreted glycoprotein, was first detected in conditioned medium of cultured human A2058 melanoma cells as an autocrine motility factor [1]. ATX belongs to the ectonucleotide pyrophosphatase/phosphodiesterase (ENPP) family and is also known as ENPP2 [1,2,3]. It was found that ATX possesses lysophospholipase D (LysoPLD) activity and mainly hydrolyzes lysophosphatidylcholine (LPC) to form lysophosphatidic acid (LPA) [4]. As a bioactive lipid molecule, LPA interacts with six different G protein-coupled receptors (LPAR_1–6_) on the cell membrane to activate the coupled G proteins (Gq, Gi and G12/13) and their downstream signaling molecules such as Rho, PLC, Ras, PI3K, and so on [5]. The ATX-LPA-LPA receptor axis participates in many physiological processes, including embryonic development, angiogenesis, and preadipocyte differentiation. When ATX or LPA receptor expression patterns are disrupted, various pathological processes can result, such as cancer [6,7], neuropathic pain [8], and fat mass expansion [9]. On the other hand, extracellular LPA is degraded to monoacylgycerol (MAG) by lipid phosphate phosphohydrolase LPPs (LPP1-3) localized on plasma membranes, which moderates the LPA effects on the activation of LPARs [10,11] (Figure 1).

## 2. ATX Structure and Isoforms

ATX protein is composed of two N-terminal somatomedin B (SMB)-like domains, a central phosphodiesterase (PDE) domain, and a C-terminal nuclease (NUC)-like domain. The PDE domain is located in the center of the peptide chain and interacts with the two SMB domains in the N-terminus and the NUC domain in the C-terminus. The PDE domain contains the active catalytic site and two zinc ions. Next to the active site, a hydrophobic lipid binding pocket can bind different LPC and LPA species. A disulfide bond joins the NUC domain and the PDE domain, and the NUC domain wrapped by a lasso maintains the rigidity of the PDE domain [12,13,14]. The N-terminal SMB domain is relatively small and enriched with cysteine, which is critical for ATX interacting with cell surface integrins [12]. The integrin binding localizes ATX activity to the cell surface, providing a mechanism to generate LPA in the vicinity of LPA receptors, achieving a highly efficient delivery of its product to the target receptors [14,15] (Figure 1).

**Figure 2:**
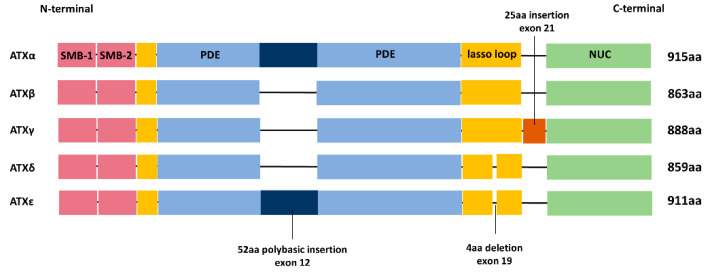
Domain structure of the major ATX isoforms. The five splice variants mainly differ by the presence or absence of sequences encoded by exons 12 and 21. Besides, ATXδ and ATXε have four amino acid deletions in exon 19.

Human ATX gene is located on chromosome 8 at position 8q24.1, which is a potential susceptibility genetic locus in cancer patients [16], containing 27 exons and 26 introns. According to different alternative splicing forms, there are five ATX isoforms distributed in different tissues (Figure 2). ATXβ is an isoform with the deletion of exons 12 and 21 enriched in human peripheral tissues. ATXγ, with the deletion of exon 12, is widely expressed in the central nervous system and brain. ATXα, in which exon 21 is deleted, is expressed at significantly lower levels than ATXβ and ATXγ in the nervous system and peripheral tissues [17,18,19,20]. In 2012, another two ATX isoforms (δ and ε) were identified. ATXδ and ATXε are novel alternative splice variants of ATX with four amino acid deletions in the L2 linker region of ATXβ and ATXα, respectively. It is found that, although all of the five ATX isoforms have the lysoPLD activity, ATXβ and ATXδ are the major and stable isoforms present in a wide range of organisms [21].

## 3. Mechanisms of ATX Expression Regulation

**Figure 3:**
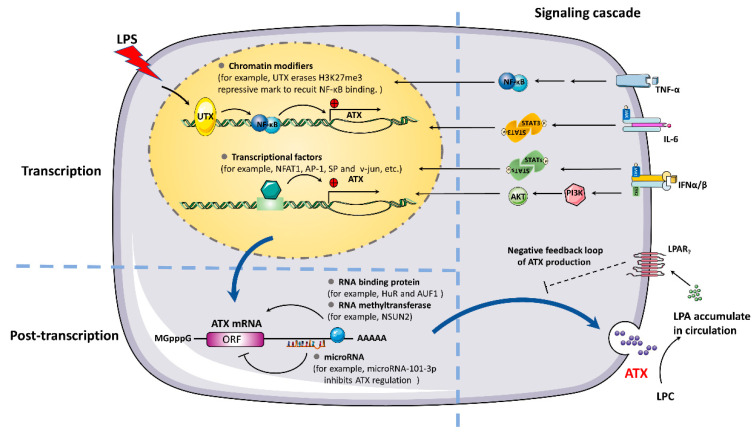
Mechanisms of ATX expression regulation. At the transcriptional level, the expression of ATX is influenced by chromatin modification and different transcriptional factors, like NFAT1, AP-1, SP, STAT3, NFκB et al. Some inflammatory cytokines, such as TNF-α, IL-6, and IFNα/β, can influence ATX expression by inducing different downstream signaling cascades. At the post-transcriptional level, RNA binding proteins (HuR and AUF1), RNA methyltransferase (NSUN2), and microRNA (miR-101-3p) bind to the 3’-UTR of ATX mRNA to influence ATX mRNA stability, nuclear export, and translation. ATX expression can be downregulated by a negative feedback loop by LPA. Moreover, the secretion of ATX is also a regulated process.

The regulation of ATX expression has a profound impact on the content of LPA in the cellular microenvironment and on the activation of the LPA receptor as well as its downstream signaling. The results of previous research indicate that ATX is regulated by complicated mechanisms at both transcriptional and post-transcriptional levels (Figure 3). It has been reported that HDAC inhibitor treatment can induce ATX expression in various cancer cells [22]. Recently, ATX promoter was characterized by H3K27me3 marks in HEK 293T cells. It was found that ATX expression levels were increased in response to lipopolysaccharide (LPS) treatment through the removal of the methyl groups of H3K27me3 by the demethylase UTX, suggesting that ATX expression is amenable to epigenetic regulation. Furthermore, a distant acting enhancer is identified as regulating ATX expression. The enhancer-mediated enrichment of interacting JMJD3-DDX21 to the ATX locus prevents R-loop formation and promotes transcript synthesis in response to LPS treatment [23]. 

There are several transcription factors involved in ATX expression. Reducing the expression of the transcription factor NFAT1 decreases ATX expression, leading to the suppression of melanoma cell growth and metastasis [24]. AP-1 and SP transcription factors act on the promoter of ATX by activating a CRE/AP-1-like element and a GA-box [25]. The transcription factor Stat3 increases the migration of breast cancer cells through the activation of ATX [26]. Michelle et al. reported that hypoxia increased hepatocellular ATX expression by HIF-1α in vitro and that hepatitis C virus infection increased ATX expression via HIF-1α to promote liver fibrosis and injury [27]. In addition, the transcription factors v-jun, c-jun, and HOXA13 all participate in the regulation of ATX at the transcriptional level [28,29,30].

In recent years, the post-transcriptional regulation of ATX expression has been identified. It is found that the RNA-binding proteins HuR and AUF1 directly bind to the ATX mRNA 3’-UTR and influence ATX mRNA stability in Colo320 human colon cancer cells and LPS-stimulated THP-1 human monocytic cells [31]. microRNA-101-3p (miR-101-3p), a well-known tumor suppressor, inhibits ATX regulation by directly targeting a conserved sequence in the ATX mRNA 3’-UTR [32]. Recently, we clarified that the RNA methyltransferase NSUN2 could methylate the 3’-UTR of ATX mRNA at cytosine 2756. Methylation by NSun2 promotes the export of ATX mRNA from the nucleus to the cytoplasm and enhances the translation of ATX [33].

Some inflammatory cytokines have been demonstrated to influence ATX expression. The proinflammatory cytokine TNFα selectively promotes ATX expression and secretion in Hep3B and Huh7 cells [34]. LPA induces the expression of IL-6, and then IL-6 stimulates the fibroblast expression of ATX, which connects the ATX/LPA and IL-6 pathways in an amplification loop in human dermal fibroblasts [35]. Our laboratory found that ATX expression in THP-1 cells was induced by treatment with TLR ligands such as LPS, CpG oligonucleotide, and poly(I:C). In response to TLR activation, ATX is induced by a type I interferon autocrine-paracrine loop through activating JAK-STAT and PI3K-AKT pathways, which is likely to enhance immune cell migration during the proinflammatory response by increasing LPA levels in the microenvironment [36]. 

ATX is a secreted glycoprotein initially synthesized as a pre-pro-enzyme with a 27-residue signal peptide at its N-terminal end. ATX secretion is dependent on the hydrophobic core sequence of the signal peptide [37]. The N-glycosylation at the amino-acids N53 and N410 of ATX is required for its secretion [38]. Our lab reported that a di-phenylalanine (Phe-838/Phe-839) motif in the human ATX C-terminal region was a transport signal essential for the ATX-p23 interaction and that ATX was selectively exported from the ER through a p23, Sec24C-dependent pathway, which could be regulated by the AKT-GSK-3β signaling [39]. 

Meanwhile, the accumulation of LPA in the circulation decreases ATX expression, which forms a negative feedback loop of ATX production [40,41]. In addition, it was found that ATX could be cleared from the circulation and degraded by liver sinusoidal endothelial cells (LSECs) through the scavenger receptor [42].

## 4. Physiological Functions of the ATX-LPA Axis

ATX distribution has been explored in the stages of embryonic development and in mature individuals. During the development of mouse embryos, the protein expression of ATX is temporal and spatial. At E8.5, ATX is mainly expressed in the anterior folds of the neural tube and the most posterior region of the midbrain. ATX expression significantly increases at the floor plate of the neural tube at E10.5. At E11.5–12.5, organs in the developing era, glands, and intestines show high ATX levels. Then, mesenchymal kidney and smooth muscle tissues exhibit high ATX levels at E13.5 and E16.5, respectively. However, ATX always shows high expression levels in choroid plexus epithelial cells from E13.5 to birth [43,44]. In healthy mature individuals, adipose tissue, the central nervous system, placenta, and lymph nodes have the highest expression levels of ATX [45,46]. In addition, in a variety of biological fluids, including blood, urine, seminal fluids, and cerebrospinal fluids, relatively high concentrations of ATX protein have been detected [47]. All these results suggest that the ATX-LPA axis may participate in the physiological and pathological processes of these organs.

To date, studies have shown that ATX plays crucial roles in embryonic development. Homozygous ATX-knockout mice show embryonic lethality with defects in the formation of blood vessels in the extraembryonic yolk sac and neural tube [48,49,50]. Heterozygous ATX-knockout mice appear to be healthy. However, the plasma LPA levels of heterozygous ATX-knockout mice are only half of those of wild-type mice [49]. This finding is consistent with a previous study showing that ATX is the main enzyme involved in LPA production in vivo. 

Because of the abundant expression of ATX in adipose tissue, many researchers have focused on the relationship between ATX and lipid metabolism. Some studies have shown that ATX expression levels are significantly increased during differentiation in 3T3-L1, 3T3-F442A, and primary preadipocytes [51,52,53]. Dusaulcy et al. reported that plasma LPA levels were reduced by 38% in mice with an adipose-specific disruption of ATX. When fed a high-fat diet, the adipocyte-specific ATX knockout mice show less insulin resistance than control mice, suggesting that ATX from adipose tissue contribute to the impaired glucose homeostasis observed in diet-induced obesity [9,54]. We found that the interleukin 6 (IL-6) family cytokines, such as IL-6, leukemia inhibitory factor, cardiotrophin-1, and ciliary neurotrophic factor, upregulated ATX expression in adipocytes through gp130. Blocking gp130 signaling suppresses ATX expression in adipocytes and improves insulin sensitivity in diet-induced obesity [53]. Growing evidences indicate that ATX is stimulated by inflammation and acts as an enhancer of inflammation in obese individuals. The adipose-specific disruption of ATX mice shows a significant decrease in IL-6, TNFα, and MCP-1 in adipose tissue and circulating plasma [54]. Incubation of adipose tissue CD8+ T cells with recombinant ATX increases the expression of CD44 and interferon, which both play predominant proinflammatory roles [54].

The ATX-LPA axis is closely related to the development of atherosclerosis [55,56,57]. As the main cause of atherosclerosis, low-density lipoproteins (LDLs), which can transform into LPC after oxidative modification, are the main sources of LPA [58]. LPA can induce endothelial cells to secrete a variety of cytokines (IL-8, IL-1β), chemokines (MCP-1, cxcl-1), and adhesion molecules (E-selectin, VCAM-1, cam-1), which mediates the recognition and adhesion of lymphocytes to endothelial cells [58,59,60]. In addition to triggering aseptic inflammation, the ATX-LPA axis can promote and deteriorate atherosclerosis by increasing endothelial permeability [61], activating vascular smooth muscle cells [62], and promoting plaque instability [63]. 

ATX also shows high expression levels in the lymphoid node and participates in lymphocyte trafficking and immune regulation. ATX can bind to lymphocytes, and LPA or ATX/LPC strongly enhances the transendothelial migration of integrin-arrested T cells across an endothelial monolayer and promotes lymphocytes to enter lymphoid organs from blood [45,64].

ATX also plays an important role in maintaining pregnancy. The serum ATX levels of pregnancy women are likely from placental trophoblasts and increase in parallel with the gestational weeks [65]. The decrease of placental ATX mRNA levels is closely associated with abnormal pregnancies [66]. After delivery, the serum ATX levels soon decrease to the nonpregnant level [67]. 

## 5. Roles of the ATX-LPA Axis in Cancers

It has been reported that ATX is highly expressed in many kinds of cancers, such as melanoma [1], glioblastoma [68], renal cancer [69], liver cancer [70], and hepatocarcinoma [71]. The ATX-LPA axis plays crucial roles in tumorigenesis and cancer cell invasion.

### 5.1. Glioblastoma Multiforme

Glioblastoma multiforme (GMB) is the most highly malignant type of brain tumor in adults. Because GMB cells are highly mobile and infiltrate normal brain parenchyma diffusely, patients with GMB have a poor prognosis and a high recurrence rate. About 26–33% of GMB patients survive more than two years in clinical trials [72], and the 5-year survival rate is only 4–5% [73].

GBM possesses a high invasiveness due to the production of LPA by ATX [68]. Yasuhiro et al. reported that GBM tissues and most brain cancer cell lines, namely SNB-78, SNB-75, SF-268, SF-539, and SF-298 cells, displayed high ATX expression levels [74]. As verified with GBM patient samples, ATX is highly expressed in glioblastoma cells and is also clearly expressed in WHO grade I, II, and III gliomas. ATX displays predominant cytoplasmic staining not only in invading cells but also in tumor core cells [68]. Oxidative stress mediates the cross talk between APE1, PKM2, and ATX and increases ATX expression levels, which stimulates the invasive potential of C6 rat and U87 human MG glioblastoma cells [75]. GBM invasion into the oligodendrocyte layer is more powerful in U87 and U251 cells, which express high ATX levels [76]. Our laboratory found that NSun2-mediated ATX mRNA methylation elevated ATX expression levels and promoted the migration of U87 cells [33]. 

Treatment with the ATX-specific inhibitor PF-8380 decreases cell migration and invasion in vivo and abrogates radiation-induced cancer neovascularization, suggesting that the inhibition of ATX may ameliorate the GBM response to radiotherapy [77,78]. The use of α-bromomethylene phosphonate LPA (BrP-LPA), an inhibitor of ATX activity and a pan-antagonist of four LPA receptors [79,80], reduced Akt phosphorylation in irradiated endothelial cell lines and decreased the survival and migration of irradiated glioblastoma cells [81]. These findings suggest that the ATX-LPA axis has a potential therapeutic relevance for GMB as a drug target.

### 5.2. Breast Cancer

Breast cancer is the most common cancer in females and constitutes 18% of all cancers in women. The estimated 5-year survival rate of breast cancer is 80% in developed countries and below 40% in developing countries [82]. A clinical study showed that serum ATX levels were significantly higher in breast cancer patients than in healthy individuals [83]. It has been reported that overexpression of ATX promotes the migration and invasion of MCF-7 and MDA-B02 cells [84,85].

In contrast to glioblastoma and melanoma cells, breast cancer cells directly produce low levels of ATX [86,87,88]. However, ATX is secreted by adjacent mammary adipose tissue and tumor-associated fibroblasts of breast cancer [89]. Breast cancer is a prototypical model of the relationship between the tumor microenvironment and ATX. Benesch et al. reported that the tumor stroma of breast cancer patients showed nearly threefold higher ATX protein levels than the normal breast stroma in healthy women [89]. Inflammatory mediators secreted by breast cancer cells, such as TNFα and IL-1β, increase ATX production in adipose tissue. Increased LPA signaling further promotes inflammatory mediator production in adipose tissue and tumors. Finally, breast cancer cells recruit ATX to bind integrin αIIbβ3 [14] and αvβ3 [90] on the cell surface to promote persistent directional cell migration [83,91,92]. 

### 5.3. Hepatocellular Carcinoma

Hepatocellular carcinoma (HCC) is the major histological type of liver cancer and the second leading cause of cancer mortality worldwide [93]. The serum ATX activity and plasma LPA levels are significantly increased in patients with liver cancer versus normal patients [94,95]. Wu et al. reported that the positive rate of ATX protein expression in HCC was 89% (34 of 38), while ATX in normal samples was 20% [34]. Hepatoma Hep3B and Huh7 cells display a higher ATX expression [34]. In mice, different hepatotoxic stimuli linked with the development of different forms of chronic liver disease are shown to stimulate hepatocyte ATX expression, leading to increased LPA levels, activation of hepatic stellate cells (HSCs), and amplification of profibrotic signals, while the hepatocyte-specific deletion of ATX attenuates HCC development [70].

### 5.4. Ovarian Cancer

Ovarian cancer is the second most common malignancy after breast cancer in women over the age of 40 years, especially in developed countries [96]. Ovarian cancer is known as a silent killer that is usually not diagnosed until vague symptoms appear when the disease has progressed to stage III or IV [97]. In ovarian cancer tissue, ATX levels were found to be at least twofold higher than in normal ovarian tissue [98,99]. In women with ovarian cancer, the activity of ATX was found to be markedly elevated in malignant ovarian ascites compared with serum and plasma [98]. Tokumura et al. revealed higher ATX levels in the peritoneal fluid than in the sera of stage III ovarian cancers, dermoid cysts, and mucinous cystadenomas [100]. The increased levels of LPA found in ascites may also be due to the release of LPA in a PLA2-dependent manner from human mesothelial cells [101]. The peritoneal fluid of ovarian carcinoma patients promotes cancer cell invasion and metastatic spread with LPA as a potentially crucial mediator. The addition of ATX inhibitor S32826 or PF8380 abolished the generation of LPA in ovarian cancer stem cells (CSCs) and resulted in the reduction of CSC characteristics, suggesting that ATX regulates the maintenance of ovarian cancer stem cells through an LPA-mediated autocrine mechanism [102].

## 6. Development and Application of ATX Inhibitors

The ATX-LPA axis plays key roles in promoting tumor migration, metastasis, invasion, and angiogenesis. Blocking the LPA formation and ATX-LPA axis signaling pathway would be an effective and important method for the treatment of cancers. Meanwhile, ATX is also seen as a potential target for treating inflammatory diseases such as pulmonary fibrosis and chronic hepatitis [103]. To date, several ATX inhibitors have reached clinical and preclinical stages of drug development.

The ATX competitive inhibitor of GLPG1690 has been in phase III clinical trials for idiopathic pulmonary fibrosis (IPF) [104,105,106]. GLPG1690 is retained in the hydrophobic pocket and hydrophobic channel of ATX to prevent the formation of LPA [105]. Recently, the effect of GLPG1690 has been studied in breast cancer. The application of GLPG1690 significantly decreased cancer cell proliferation and enhanced radiotherapy-induced apoptosis in a mouse model of breast cancer [107].

PF-8380 is another potent inhibitor of ATX that can occupy the orthosteric site directly and mimic LPC substrate binding. Inhibition of ATX by PF-8380 leads to suppressed tumor invasion and enhances radio-sensitization in human and murine glioblastoma cell lines [77]. The application of PF-8380 not only decreases high fat diet-induced cardiac hypertrophy, dysfunction, and inflammatory responses by reducing circulating LPA levels in obese mice [108] but also attenuates bleomycin-induced pulmonary fibrosis [109]. 

ONO-8430506, a new ATX inhibitor, decreases plasma ATX activity. ONO-8430506 decreases initial breast cancer cell growth and subsequent lung metastatic nodules by 60% compared with vehicle-treated mice [110]. The use of ONO-8430506 decreases the levels of 16 inflammatory mediators in thyroid cancer and is associated with a 50–60% decrease in tumor volume [111].

S32826 is a nanomolar inhibitor of ATX that lacks activity in vivo. However, it is widely used for in vitro studies [112]. Our laboratory found that TSA-induced ATX protected cancer cells against TSA-induced apoptosis by producing LPA through its lyso-PLD activity, which could be reversed by S32826 [22]. 

HA155 and HA130 are boronic acid-based compounds that inhibit ATX by selectively binding to its catalytic threonine and inhibiting ATX binding with LPC [12,113,114]. 

RB011 is a DNA aptamer that can specifically bind to the active site of ATX and inhibit LPC access to the hydrophobic binding pocket [115]. However, it has been indicated that RB011 can also inhibit ENPP1 activity [115].

## 7. Future Works

The expression of ATX is characteristic of spatiotemporal specificity and tissue specificity. ATX expression is regulated in many ways, including regulation at the transcription, post-transcription, and secretion levels. According to the existing results, ATX is highly expressed in various cancer cells, and the ATX-LPA axis is of great significance in oncogenesis and cancer progression. So far, the mechanism of ATX expression in cancer cells is still not fully clear and needs to be further clarified. The modulation of the activity and localization of extracellular ATX as well as the degradation of ATX are attractive subjects for future study. ATX is regarded as a cancer therapy target, and several ATX inhibitors have been developed and applied in preclinical trial. These ATX inhibitors may be used in the clinical treatment of cancer in the future.
